# Distributional Cost-Effectiveness Analysis: A Tutorial

**DOI:** 10.1177/0272989X15583266

**Published:** 2015-04-23

**Authors:** Miqdad Asaria, Susan Griffin, Richard Cookson

**Affiliations:** Centre for Health Economics, University of York, York, UK (MA, SG, RC)

**Keywords:** cost-effectiveness analysis, economic evaluation, efficiency, equality, equity, fairness, health distribution, health inequality, inequality measures, opportunity cost, social value judgments, social welfare functions, tradeoff

## Abstract

Distributional cost-effectiveness analysis (DCEA) is a framework for incorporating health inequality concerns into the economic evaluation of health sector interventions. In this tutorial, we describe the technical details of how to conduct DCEA, using an illustrative example comparing alternative ways of implementing the National Health Service (NHS) Bowel Cancer Screening Programme (BCSP). The 2 key stages in DCEA are 1) modeling social distributions of health associated with different interventions, and 2) evaluating social distributions of health with respect to the dual objectives of improving total population health and reducing unfair health inequality. As well as describing the technical methods used, we also identify the data requirements and the social value judgments that have to be made. Finally, we demonstrate the use of sensitivity analyses to explore the impacts of alternative modeling assumptions and social value judgments.

## Introduction

When designing and prioritizing interventions, health care decision makers often have concerns about reducing unfair health inequality as well as improving total population health. However, the economic evaluation of such interventions is typically conducted using methods of cost-effectiveness analysis (CEA), which focus exclusively on maximizing total population health. These standard methods of CEA do not provide decision makers with information about the health inequality impacts of the interventions evaluated, or the nature and size of any tradeoffs between improving total population health and reducing unfair health inequality.

To address these shortcomings, we have developed a framework for incorporating health inequality impacts into CEA, which we call *distributional cost-effectiveness analysis* (DCEA).^1^ DCEA is suitable for health sector decisions concerning the design and prioritization of any type of health care intervention with an explicit health inequality reduction objective—potentially including treatments as well as preventive health care such as programs of health promotion, screening, vaccination, case finding, primary and secondary prevention of chronic disease, and so on. However, like standard CEA, it focuses exclusively on health benefits and opportunity costs falling on the health sector budget. DCEA therefore does not provide a fully general framework of distributional economic evaluation for the health and income inequality impacts of cross-government public health programs with important nonhealth benefits and opportunity costs falling outside the health sector budget.

The DCEA framework has 2 main stages: 1) modeling social distributions of health associated with each intervention, and 2) evaluating social distributions of health. The main steps in the modeling stage are

1a. estimating the baseline health distribution;
1b. modeling changes to this baseline distribution due to the health interventions being compared, allowing for the distribution of opportunity costs from additional resource use; and
1c. adjusting the resulting modeled health distributions for alternative social value judgments about fair and unfair sources of health variation.

And the main steps in the evaluation stage are

2a. using the estimated distributions to quantify the change in total population health and unfair health inequality due to each intervention;
2b. ranking the interventions based on dominance criteria; and, finally,
2c. analyzing any tradeoffs between improving population health and reducing unfair health inequality, allowing for alternative specifications of the underlying social welfare function.

We have previously applied the DCEA framework to analyze 4 possible options for promoting increased uptake of the National Health Service (NHS) Bowel Cancer Screening Programme (BCSP) in England.^2^ In this tutorial, we work through this applied example to describe the key steps in conducting a DCEA.

The BCSP is a biennial self-test-based screening program targeted at 60–74 year olds that aims to detect and treat colorectal cancer (CRC) early, and it has been shown to reduce CRC-related mortality risk by a substantial proportion. Individuals in the relevant age range are sent a guaiac fecal occult blood test (gFOBT) kit in the mail and are expected to complete the test by collecting 3 stool samples during a period of a few days and post them back for laboratory analysis. Those individuals testing positive are invited for further diagnostic testing (follow-up colonoscopy) and, when appropriate, treatment.

Analysis of the BCSP pilots and early data from the rollout of the BCSP have indicated large variations in uptake of the screening program patterned by the social variables of area deprivation, sex, and ethnicity. This variation in uptake can be modeled to estimate its impact on mortality and morbidity for the different socioeconomic subgroups in the population, and hence to describe the impact of the screening program on both the average level of health and the social distribution of health in the population.

## Methods

### Stage A: Modeling Social Distributions of Health

#### Estimating the baseline health distribution

The first step in DCEA is to describe the baseline distribution of health, taking into account variation in both length and health-related quality of life. This baseline distribution will need to include the full general population, and not just the population of recipients of the intervention. This is for 2 reasons. First, the full general population is typically the relevant population for characterizing policy concerns with health inequality. Second, within the context of a national, budget-constrained system such as the NHS, additional resources used by recipients of an intervention will displace activities that could have been provided to anyone within the full general population.

This baseline distribution of health should be able to describe variation in health among multiple different subgroups in the population as defined by relevant population characteristics, allowing for the correlation structure among these various characteristics. The relevant population characteristics include not only dimensions of direct equity concern (e.g., income and ethnicity) but also characteristics that are necessary to estimate expected costs and effects and that may generate further equity concern (e.g., sex). The latter of these is standard for any cost-effectiveness analysis (CEA), whereas the former we discuss further throughout this tutorial. The health metric we use in this context is quality-adjusted life expectancy (QALE) at birth, although other suitable health metrics could also be used—such as disability-adjusted life expectancy at birth or age-specific QALE—as long as they are measured on an interpersonally comparable ratio scale suitable for use within a CEA.

The population characteristics of interest in this case study—those by which a substantial variation in uptake of the BCSP was observed—are sex, area-level deprivation, and area-level ethnic diversity. The first step in estimating our population QALE distribution is to estimate life expectancy (LE), according to each of these characteristics. Area-level deprivation in the BCSP evaluation studies was measured based on index of multiple deprivation (IMD 2004) quintile groups, and area-level ethnic diversity was based on the percentage of people in the area originating from the Indian Subcontinent, again split into quintile groups.^3^ National statistics data are available by sex and deprivation level/social class, but are not available by our particular measure of ethnic diversity. We therefore did not include correlations with ethnic diversity in our estimation of the baseline health distribution and instead, for the purposes of the analysis, assumed its distribution is independent of deprivation and sex.

**Figure 1:**
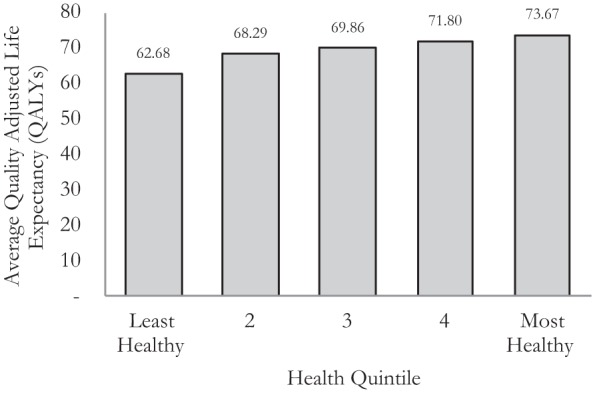
Baseline health distribution.

A full description of how the baseline health distribution was calculated can be found in the appendix. A summary of this QALE distribution by health quintile is shown in Figure 1. This forms the baseline health distribution that we will use in our analysis.

#### Estimating the distribution of health changes due to the interventions

To evaluate changes in the baseline health distribution that could be attributed to the use of alternative interventions, it is necessary to know how the costs and effects of the intervention differ between the relevant subgroups, and how the opportunity costs of any change in resource use differ by those same subgroups.

Having estimated a baseline health distribution, we next turn to modeling how this health distribution is affected by the BCSP and alternative ways of promoting increased uptake of the BCSP. We do this by using an existing cost-effectiveness model of the BCSP that simulates the natural history of CRC and the impact of screening and treatment on this natural history.^4,5^ We adapt the model to look at the distributional health impacts of 4 different screening strategies:

1. *No screening*: the baseline social distribution of health
2. *Standard screening*: as implemented in the BCSP
3. *Targeted reminder*: screening plus a targeted enhanced reminder letter (personal general practitioner [GP] signed letter and tailored information package) sent only to those living in the most income-deprived small areas (IMD4 and IMD5) as well as to those living in areas with the highest proportion of inhabitants from the Indian Subcontinent (IS5)
4. *Universal reminder*: screening plus a universal basic reminder letter (sending a GP-endorsed reminder letter to all eligible patients)

Impacts are first estimated by subgroup and then combined to evaluate the impact of the screening strategies on the overall social distribution of health.

There are a number of parameters in the model that can vary by subgroup, including:

1. *Disease prevalence, severity, mortality rate, and natural history*: We assume in our case study that bowel cancer–specific parameters are constant across our population subgroups. The evidence available^6^ broadly supports this assumption, although more detailed data at the subgroup level would be required to validate this assumption.
2. *Uptake of the intervention*: The impact of gFOBT uptake by subgroup is the key difference between the various implementations of the screening program. We discuss in detail in this article how this parameter is estimated for each subgroup. We also estimate the uptake of follow-up colonoscopy by subgroup for those people who are invited back for further investigation after being screened.
3. *Direct costs associated with the intervention*: We assume the direct costs related to treating a given stage of bowel cancer do not vary by subgroup (although the chance of incurring these costs and the screening-related costs by subgroup may vary under the different implementations of the screening program). This seems to be a plausible assumption in the absence of more detailed cost data at the subgroup level.
4. *Opportunity costs from displaced activities*: Opportunity costs are in the base case analysis assumed to be shared equally among all population subgroups; this assumption is explored in sensitivity analyses discussed later in this tutorial.
5. *Other-cause mortality*: We use the mortality rates by subgroup in the same way as discussed when deriving the baseline health distribution. In calculating these rates, we remove bowel cancer–specific mortality (assuming this is constant across subgroups) and apply this separately in the model.

*Quality adjustment of health gains to reflect morbidity*: We apply the subgroup-specific adjustments to quality-adjust health gains resulting from the screening program in a similar manner to that applied to estimate the baseline health distribution. The population QALE distribution under no screening corresponds to our baseline health distribution as calculated in the previous section. In our analysis of the BCSP, we include an additional variable—area-level proportion of population from the Indian Subcontinent (IS)—which we were unable to incorporate into our estimation of the baseline health distribution. We assume that this IS variable is distributed independently of IMD and sex, and that it has no independent effect on baseline QALE (i.e., subgroups are adjusted for other-cause mortality and quality adjusted only according to their IMD and sex, and these adjustments are not affected by their IS status). We next adjust the BCSP uptake parameters by subgroup. Table 1 shows logistic regression results looking at gFOBT uptake in the 3 rounds of the BCSP pilot.^3^ We use these data in combination with the proportion of invitees in each category by variable, also reported in the pilot evaluation, to get weighted average odds ratios (ORs) for uptake that can be applied in the model.

**Table 1:** Regression Results of gFOBT Uptake from Evaluation of BCSP Pilot

		Adjusted OR (95% CI)
Age (years)	57–59	1
	60–64	1.13 (1.11–1.16)
	65–69	1.25 (1.22–1.28)
Sex	Male	1
	Female	1.38 (1.35–1.40)
Pilot round	1	1
	2	0.77 (0.76–0.80)
	3	0.82 (0.81–0.84)
Deprivation category (IMD)	Q1 (Least deprived)	1
	Q2	0.84 (0.81–0.87)
	Q3	0.70 (0.68–0.72)
	Q4	0.55 (0.54–0.57)
	Q5 (Most deprived)	0.37 (0.35–0.38)
% Indian Subcontinent	Q1–4	1
	Q5 (Highest %)	0.86 (0.84–0.89)

BCSP, National Health Service (NHS) Bowel Cancer Screening Programme; CI, confidence interval; gFOBT, guaiac fecal occult blood test; IMD, index of multiple deprivation; OR, odds ratio.

These ORs are applied to a baseline rate of uptake reported in the third-round pilot, in which males in the youngest age group, living in the most deprived areas and with the highest proportion of people from the Indian Subcontinent, had an uptake probability of 34%. For example, to calculate the uptake probability for a woman of any age across all rounds of the pilot, living in the least deprived areas and with the least numbers of people from the Indian Subcontinent, we can use the following calculation:

$$\begin{array}{l} OR=0.34/(1-0.34)*(1.38/0.82)*1.13*0.86*\\ (1/0.37)*(1/0.86)=2.71\\ P=OR/(1+OR)=0.73 \end{array}$$

A similar regression analysis was reported analyzing the effect of these same variables on the uptake of follow-up colonoscopy. Data were also published in the pilot study evaluation regarding the numbers of people in each category for each variable in the study. However, cross-tabulations or correlations between the variables were not available, and we therefore assumed that each variable was independently distributed to calculate the proportion of the population in each subgroup. Table 2 shows our calculated gFOBT uptake, the follow-up colonoscopy uptake, and the proportion of the population by each subgroup.

**Table 2:** gFOBT Uptake, Follow-Up Colonoscopy Uptake, and Proportion of Population by Subgroup

Sex	% Indian Subcontinent	Deprivation (IMD quintile)	gFOBT Uptake (%)	Colonoscopy Uptake (%)	Population Proportion (%)
Male	Q1–4	Q1 (Least deprived)	66	86	6
		Q2	62	84	9
		Q3	58	80	10
		Q4	52	79	8
		Q5 (Most deprived)	42	77	6
	Q5 (Highest)	Q1 (Least deprived)	63	87	1
		Q2	59	85	2
		Q3	54	81	3
		Q4	48	79	2
		Q5 (Most deprived)	38	75	2
Female	Q1–4	Q1 (Least deprived)	73	85	6
		Q2	70	83	9
		Q3	66	79	10
		Q4	60	77	8
		Q5 (Most deprived)	50	76	6
	Q5 (Highest)	Q1 (Least deprived)	70	86	1
		Q2	66	83	2
		Q3	62	79	3
		Q4	56	78	2
		Q5 (Most deprived)	46	76	2

gFOBT, guaiac fecal occult blood test; IMD, index of multiple deprivation.

Using these parameters in the model provides the total costs and health gains due to the BCSP under the standard screening approach.

We next turn to modeling the remaining 2 implementations of the screening program. Both implementations augment the standard screening program with additional reminders. We derive the indicative estimates of costs and impacts on screening uptake of these reminder strategies from similar interventions studied in the screening literature,^7,8^ applying plausible exchange rates and inflation rates to the figures to get costs, and assuming all subgroups receiving the interventions have equal additive increases in uptake. The values used in the model for costs and impacts on gFOBT uptake for each of the strategies are given in Table 3.

**Table 3:** Costs and Impact on gFOBT Uptake of Reminder Strategies

Strategy	Cost per Recipient	Increase in gFOBT Uptake per Recipient
Universal reminder	£3.50	6%
Targeted reminder	£7.00	12%

gFOBT, guaiac fecal occult blood test.

To estimate total costs and health effects, the model is evaluated for a representative cohort of the population—in our case, a cohort of 1 million 30 year olds, as was used in the original analysis of the BCSP in the model we inherited. The size of each subgroup is given by the population proportions calculated in Table 2. We sum the costs across all subgroups, and convert these to health opportunity costs using a threshold value of £20,000 per quality-adjusted life year (QALY). These health opportunity costs are then apportioned equally to each individual in the population, allowing the model to characterize net health gains in each subgroup. For example, the total costs for the standard screening program during the lifetime of the cohort of 1 million patients came to £72 million. Converting this to health opportunity costs at the rate of £20,000 per QALY gives us 3600 QALYs of health opportunity costs. Women who live in areas with a low percentage of the population from the Indian Subcontinent (IS Q1–4), and who also fall within deprivation quintile IMD Q3, make up 10% of the population. So we allocate 10% of this total health opportunity cost to them (i.e., 360 QALYs). This is then subtracted from the total health gains due to the BCSP in this subgroup to give the net health effect of the BCSP on this subgroup.

The assumption of equally distributed opportunity costs is convenient but not evidence based. So we explore alternative assumptions in sensitivity analysis, focusing on 2 extreme cases in which all opportunity costs are allocated to the least healthy and the healthiest subgroups, respectively.

The additional parameters that we have added to the model are assigned standard distributions by variable type, and their mean and standard error values are used to generate suitable random draws for these variables in the probabilistic sensitivity analysis (PSA). Details of how these additional variables are dealt with in the PSA are given in Table 4. All the results presented are produced by running the model probabilistically and averaging more than 1000 iterations of the model.

**Table 4:** Distributions and Parameter Values Used in PSA for Additional Parameters Added to the Model

Parameter	Explanation
gFOBT and colonoscopy uptake	Uncertainty on these calculated in PSA assuming ln(OR) distributed normally. The variance covariance matrices for the uptake regressions were not available to us, so we drew each coefficient independently and combined to create uptake probabilities.
Mortality rates	Adjusted for uncertainty by the underlying model.
Quality adjustment	Used β distribution with the mean and standard error values as reported in the UK EQ-5D norms.
Cost of reminders	As no data were given on the uncertainty, we assume a 10% standard error and used this to draw values from the appropriate γ distributions.
Impact of reminders on uptake	Reported mean and standard error values used to draw from the appropriate β distributions.

gFOBT, guaiac fecal occult blood test; OR, odds ratio; PSA, probabilistic sensitivity analysis.

**Figure 2:**
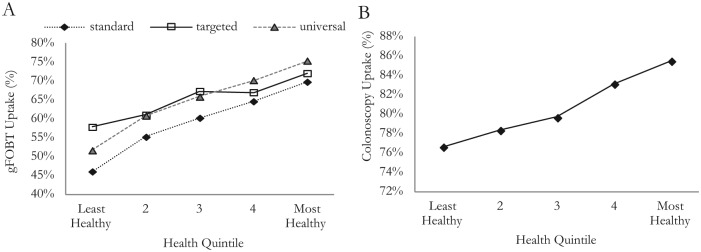
(A) Guaiac fecal occult blood test (gFOBT) uptake distribution by strategy; and (B) colonoscopy uptake distribution.

**Figure 3:**
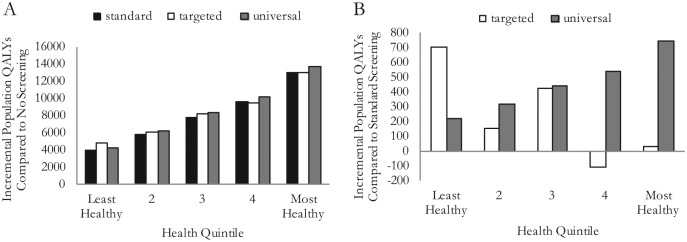
(A) Health compared to no screening (per million of population invited for screening); and (B) health compared to standard screening (per million of population invited for screening).

The resulting health distributions estimated for each screening implementation are described in Figures 2 and 3 and Table 5. Figure 2A shows the gFOBT uptake by health quintile for each strategy, and Figure 2B shows the colonoscopy uptake by health quintile. QALE for each subgroup calculated from our adjusted model is given in Table 5, and these are presented for our cohort by health quintile in Figure 3A and Figure 3B, allowing us to better appreciate the relative impacts of the strategies.

**Table 5:** QALE Distribution by Subgroup Under Each Strategy

			QALE			
Sex	% Indian Subcontinent	Deprivation (IMD quintile)	Baseline	Standard	Targeted	Universal
Male	Q1–4	Q1 (Least deprived)	72.16	72.21	72.20	72.21
		Q2	70.48	70.52	70.52	70.52
		Q3	69.09	69.12	69.12	69.13
		Q4	66.61	66.63	66.63	66.63
		Q5 (Most deprived)	60.22	60.24	60.24	60.24
	Q5 (Highest)	Q1 (Least deprived)	72.16	72.20	72.21	72.21
		Q2	70.48	70.52	70.52	70.52
		Q3	69.09	69.12	69.13	69.12
		Q4	66.61	66.63	66.63	66.63
		Q5 (Most deprived)	60.22	60.23	60.24	60.23
Female	Q1–4	Q1 (Least deprived)	74.84	74.91	74.91	74.92
		Q2	73.10	73.16	73.16	73.17
		Q3	71.77	71.82	71.81	71.82
		Q4	69.19	69.23	69.24	69.23
		Q5 (Most deprived)	63.17	63.20	63.20	63.20
	Q5 (Highest)	Q1 (Least deprived)	74.84	74.91	74.92	74.91
		Q2	73.10	73.16	73.17	73.16
		Q3	71.77	71.81	71.82	71.82
		Q4	69.19	69.23	69.24	69.23
		Q5 (Most deprived)	63.17	63.20	63.20	63.20
Overall average			69.260	69.300	69.301	69.302

IMD, index of multiple deprivation; QALE, quality-adjusted life expectancy.

#### Adjusting for social value judgments about fair and unfair sources of inequality

The distributions of health estimated thus far represent all variation in health in the population. However, some variation in health may be deemed “fair,” or at least “not unfair,” perhaps because it is due to individual choice or unavoidable bad luck. In such cases, the health distributions should first be adjusted to only include health variation deemed “unfair” before measuring the level of inequality. Social value judgments need to be made about whether health variation associated with each of the population characteristics is deemed fair. In our example, we have 3 variables to consider: sex, IMD, and ethnicity. We might make the value judgment that differences in health due to sex are fair, whereas differences in health due to IMD and ethnicity are unfair—this is 1 of 8 possible value judgments that we can make on fairness in this example. One way of adjusting our modeled health distributions for this value judgment is by using direct standardization.^9^ To do this, we run a regression on our QALE distribution weighting the subgroups by the proportion of the population they represent to find the association between each variable and QALE. An example of such a regression is given in Table 6. We then use reference values for those variables deemed fair (i.e., sex in this case) while leaving the other variables to take the values they have in the relevant subgroups and predict out an adjusted QALE distribution. In this example, we use male as the reference value for sex and predict out the QALE distribution as shown in Table 7. This distribution represents only the variation in health deemed unfair by the social value judgment made. Reference values used in the adjustment process are typically population averages for continuous variables, whereas for categorical variables the most commonly occurring category is typically used with sensitivity analysis performed on the impact of alternative choices of reference category.

**Table 6:** Fairness Adjustment Regression

	Coefficient (SE)
Constant	74.92
	(4.37E-05)
IS Q1–4	−0.004
	(2.56E-05)
Male	−2.708
	(5.47E-05)
IMD Q2	−1.75
	(4.91E-05)
IMD Q3	−3.097
	(4.84E-05)
IMD Q4	−5.675
	(5.02E-05)
IMD Q5	−11.71
	(5.33E-05)
Male*IMD Q2	0.065
	(6.95E-05)
Male*IMD Q3	0.015
	(6.84E-05)
Male*IMD Q4	0.104
	(7.10E-05)
Male*IMD Q5	−0.259
	(7.532E-05)

IMD, index of multiple deprivation; IS, Indian Subcontinent; SE, standard error.

**Table 7:** Fairness Adjusted Health Distribution Reference Sex = Male

			QALE	
Sex	% Indian Subcontinent	Deprivation (IMD quintile)	Targeted	Targeted Adjusted
Male	Q1–4	Q1 (Least deprived)	72.20	72.20
		Q2	70.52	70.52
		Q3	69.12	69.12
		Q4	66.63	66.63
		Q5 (Most deprived)	60.24	60.24
	Q5 (Highest)	Q1 (Least deprived)	72.21	72.21
		Q2	70.52	70.52
		Q3	69.13	69.13
		Q4	66.63	66.63
		Q5 (Most deprived)	60.24	60.24
Female	Q1–4	Q1 (Least deprived)	74.91	72.20
		Q2	73.16	70.52
		Q3	71.81	69.12
		Q4	69.24	66.63
		Q5 (Most deprived)	63.20	60.24
	Q5 (Highest)	Q1 (Least deprived)	74.92	72.21
		Q2	73.17	70.52
		Q3	71.82	69.13
		Q4	69.24	66.63
		Q5 (Most deprived)	63.20	60.24

IMD, index of multiple deprivation; QALE, quality-adjusted life expectancy.

### Stage B: Evaluating Social Distributions of Health

#### Comparing interventions in terms of total health and unfair health inequality

Once we have estimated the appropriate health distributions, we can then go on to characterize the distributions in terms of the twin policy goals of improving total health and reducing health inequality. One useful piece of information for decision makers produced at this step of the analysis is the size of the health opportunity cost of choosing an intervention that reduces health inequality—this is simply the difference in total health between the intervention and a comparator. However, this step of the analysis can also go further than that by providing information about the size of the reduction in health inequality, in terms of the difference in 1 or more suitable inequality indices between the intervention and a comparator. The selection of appropriate inequality indices requires further value judgments about the nature of the inequality concern. There are a number of commonly used indices to measure inequality that can be broadly grouped into those measuring relative inequality (scale-invariant indices), those measuring absolute inequality (translation invariant), and those measuring health poverty or shortfall from a reference value. If there is no clear choice of inequality measure, it may be preferable to calculate a range of alternative measures.

Table 8 shows the results of calculating a range of relative and absolute inequality measures for the QALE distributions associated with our 4 screening strategies. A higher value for each measure indicates a higher level of inequality between the healthiest and the least healthy.

**Table 8:** Inequality Measures Calculated for 4 Screening Strategies

Relative Inequality Indices	No Screening	Standard	Targeted Reminder	Universal Reminder
Relative gap index (ratio)	0.17527*	0.17592	0.17586	0.17596
Relative index of inequality	0.18607*	0.18674	0.18668	0.18678
Gini index	0.03101*	0.03112	0.03111	0.03113
Atkinson index (ϵ = 1)	0.00171*	0.00172	0.00172	0.00172
Atkinson index (ϵ = 7)	0.01330*	0.01337	0.01337	0.01338
Atkinson index (ϵ = 30)	0.06253*	0.06281	0.06279	0.06283
Absolute Inequality Indices	No Screening	Standard	Targeted Reminder	Universal Reminder
Absolute gap index (range)	10.98604*	11.03064	11.02726	11.03325
Slope index of inequality	12.88747*	12.94123	12.93691	12.94438
Kolm index (α = 0.025)	0.20281*	0.20430	0.20416	0.20439
Kolm index (α = 0.1)	0.87801*	0.88429	0.88371	0.88467
Kolm index (α = 0.5)	4.56391*	4.58739	4.58587	4.58883

α = 0.025, low absolute inequality aversion; α = 0.5, high absolute inequality aversion; ϵ = 1, low relative inequality aversion; ϵ = 30, high relative inequality aversion.

* The most equal strategy.

#### Ranking interventions using dominance rules

The first step in comparing distributions is looking to commonly used distributional dominance rules, because these allow strategies to be ranked with minimal restriction to the form of the underlying social welfare function. In terms of standard economic dominance rules, we can note from Table 5 that no screening and standard screening are strictly dominated in the space of QALE by the universal reminder strategy—that is, the no sex-IMD-ethnicity subgroup is less healthy, and at least 1 subgroup is healthier. However, this rule does not account for the level of inequality. When ranking distributions based on mean health and the level of health inequality, it is possible to use alternative economic dominance rules provided by Atkinson^10^ and Shorrocks.^11^ These dominance rules apply when mean health is higher and inequality is lower for almost any measure of inequality. Both rules are based around the Lorenz curve,^12^ a tool to analyze relative inequality constructed for health distributions by ordering the population from least healthy to most healthy and plotting the cumulative proportion of population health against the cumulative proportion of the population. Regarding Atkinson’s theorem tests for Lorenz dominance between distributions, this means that the Lorenz curves for the distributions do not cross, and the more equal distribution has at least as much mean health as the less equal distribution. In other words, a distribution is dominated if it has higher inequality and the same or lower amount of mean health. On these criteria, the standard screening strategy is dominated by the targeted reminder. Shorrocks’ theorem tests for generalized Lorenz dominance, wherein the Lorenz curve is multiplied by the mean health. A distribution is dominated if the generalized Lorenz curve lies wholly below that of an alternative intervention. Under this criterion, both the targeted and universal reminder strategies dominate the no-screening option. This leaves us to compare the universal-reminder and targeted-reminder strategies. Although the universal reminder produces a higher average QALE overall and benefits the less deprived quintile groups more, the targeted reminder is the more equal strategy on every measure listed in Table 8 and benefits the most deprived quintile groups more. In our example, the generalized Lorenz curves for these 2 distributions cross, and hence we cannot use Shorrocks’ theorem to rank the distributions.

#### Analyzing tradeoffs between total health and health inequality using social welfare indices

Having used distributional dominance to eliminate no screening and standard screening, to rank the remaining 2 strategies it is necessary to specify more fully an underlying social welfare function. A number of alternative social welfare indices have been proposed that could be used to characterize the dual objectives of increasing total health and reducing health inequality. A common feature of such functions is the need to specify the nature of and level (or value) of inequality aversion. The inequality aversion parameters in these functions describe the tradeoff between total health and the level of health inequality (i.e., the amount of total health that a decision maker would be willing to sacrifice to achieve a more equal distribution). These inequality aversion parameters are difficult to interpret on a raw scale. A more intuitive scale can be provided by combining a specific value of the parameter with a specific health distribution to derive the *equally distributed equivalent* (EDE) level of health. The difference between the mean level of health in that distribution and the EDE level of health then represents the average amount of health per person that one would be willing to sacrifice to achieve full equality in health, given that specific value of inequality aversion.

In this example, we will use 2 social welfare indices closely linked to the dominance rules applied above: the Atkinson index^10^ to evaluate the distributions in terms of relative inequality, and the Kolm index^13^ to evaluate the distributions in terms of absolute inequality. The EDE for these social welfare indices can be calculated as follows using the inequality aversion parameters ε and α, respectively:

Atkinson social welfare index:

$$h_{\mathrm{ede}}={\left[\frac{1}{n}\sum_{i=1}^{n}{\left[h_{i}\right]}^{1-\varepsilon }\right]}^{\frac{1}{1-\varepsilon }}$$

Kolm social welfare index:

$$h_{\mathrm{ede}}=-\left(\frac{1}{\alpha }\right)\log\left(\frac{1}{n}\sum_{i=1}^{n}e^{-\alpha h_{i}}\right)$$

**Figure 4:**
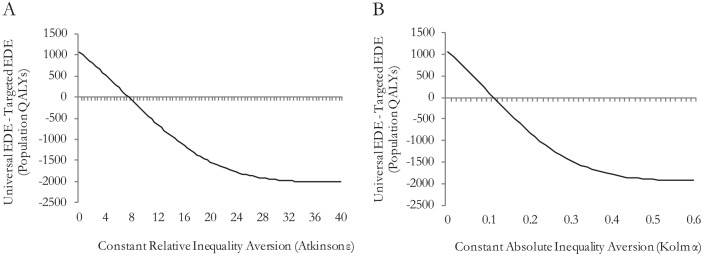
(A) Sensitivity to level of relative inequality aversion; and (B) sensitivity to level of absolute inequality aversion.

Figure 4A and Figure 4B show the difference in EDE health between the 2 strategies across different levels of inequality aversion for the relative and absolute social welfare indices, respectively. With zero inequality aversion, the EDE represents the mean health, and we see that the universal strategy offers 1000 more population QALYs compared to the targeted strategy. For inequality aversion levels greater than ε = 8 and α = 0.12, the targeted strategy would be preferred, implying that the decision maker would be willing to sacrifice those 1000 population QALYs to achieve the lower level of inequality.

Recent work on eliciting these inequality aversion parameters from members of the general public in England^14^ estimates an Atkinson ε parameter of about 10.95 (95% confidence interval [CI]: 9.23–13.54) and a Kolm α parameter of about 0.15 (95% CI: 0.13–0.19).

### Sensitivity Analysis

There are a number of sensitivity analyses we can run to explore the impact of making alternative assumptions in our modeling on our choice of preferred strategy. Tables 9 and 10 present the results, respectively, of exploring 1) the impacts of alternative assumptions around the distribution of opportunity costs, and 2) the impacts of alternative social value judgments about which inequalities are considered unfair.

**Table 9:** Sensitivity to Alternative Opportunity Cost Distributions

	All Opportunity Cost Borne by Least Healthy Subgroup				All Opportunity Cost Borne by Healthiest Subgroup	
Social Welfare Indices	No Screening	Standard	Targeted Reminder	Universal Reminder	Targeted Reminder	Universal Reminder
Mean health	69.25969	69.30006	69.30127	69.30233*	69.30127	69.30233*
Atkinson EDE (ϵ = 1)	69.14152	69.18056	69.18147	69.18252*	69.18286	69.18373*
Atkinson EDE (ϵ = 7)	68.33888	68.36800*	68.36610	68.36734	68.37799*	68.37769
Atkinson EDE (ϵ = 30)	64.92865*	64.91468	64.89302	64.89892	64.95627*	64.95350
Kolm EDE (α = 0.025)	69.05688	69.09486	69.09556	69.09660*	69.09793	69.09866*
Kolm EDE (α = 0.1)	68.38168	68.41112*	68.40958	68.41074	68.42046*	68.42020
Kolm EDE (α = 0.5)	64.69578*	64.68086	64.65951	64.66532	64.72148*	64.71879

EDE, equally distributed equivalent.

* The strategy yielding the highest social welfare.

**Table 10:** Sensitivity to Alternative Social Value Judgments

Social Value Judgment			Preferred Strategy Based on Social Welfare Index					
IMD	Ethnic Diversity	Sex	Atkinson EDE (ϵ = 1)	Atkinson EDE (ϵ = 7)	Atkinson EDE (ϵ = 30)	Kolm EDE (α = 0.025)	Kolm EDE (α = 0.1)	Kolm EDE (α = 0.5)
Fair	Fair	Fair	U	U	U	U	U	U
Fair	Unfair	Fair	U	U	U	U	U	U
Fair	Fair	Unfair	U	U	U	U	U	U
Fair	Unfair	Unfair	U	U	U	U	U	U
Unfair	Fair	Fair	U	U	T	U	U	T
Unfair	Unfair	Fair	U	U	T	U	U	T
Unfair	Fair	Unfair	U	U	T	U	U	T
Unfair	Unfair	Unfair	U	U	T	U	U	T

EDE, equally distributed equivalent; IMD, index of multiple deprivation; T, targeted reminder; U, universal reminder.

We could also perform additional sensitivity analyses, including exploring alternative ways that the reminder strategies might affect the different population subgroups (e.g., having constant proportional effects rather than constant absolute effects) and testing for alternative underlying distributions of CRC mortality, incidence, and severity.

## Discussion

DCEA is a framework for incorporating health inequality concerns into the cost-effectiveness analysis of health care interventions. It aims to help cost-effectiveness analysts provide decision makers with useful quantitative information about the health inequality impacts of health care interventions, and the nature and size of tradeoffs between the dual objectives of improving total health and reducing health inequality. It also aims to help cost-effectiveness analysts accommodate different value judgments about health inequality made by different decision makers and stakeholders.

Social value judgments about health inequality are complex, context dependent, and contestable. For this reason, DCEA does not prescribe in advance any particular set of social value judgments about health inequality. A number of social value judgments need to be made when implementing the DCEA framework, in particular regarding which dimensions of inequality are deemed unfair and the nature and strength of inequality aversion. The framework makes these social value judgments explicit and transparent, and lends itself well to checking the sensitivity of conclusions based on alternative plausible social value judgments. DCEA thus aims to provide decision makers with useful quantitative information about health inequality effects that can help to inform a deliberative decision-making process, by showing how different social value judgments might lead to different conclusions. Empirical work to estimate the nature and level of societal inequality aversion implicit in current health care allocation decisions would be useful in validating and complementing estimates of the inequality aversion levels emerging from value elicitation exercises conducted on members of the general public in England.^14^ This work would be analogous to the recent work that has been done to generate empirical estimates of the cost-effectiveness threshold.^15^

DCEA is intended to be a general and flexible analytical framework that allows a diverse range of specific methods and techniques to be applied at different stages of the analysis. In particular, the evaluation stage can in principle use any kind of equity weighting and/or multicriteria decision analysis to analyze tradeoffs between improving total health and reducing health inequality, and it is not restricted to application of the specific Atkinson and Kolm social welfare functions described in this tutorial.

We have seen in this tutorial that DCEA is demanding in terms of data, but feasible to implement in a real-world context through creative application of the standard tools of economic analysis. The data and methods we have used are inevitably partial and crude in many respects, and it is our hope that the underpinning data and technical methods will be improved and refined throughout the years. Although the framework and methods involved may seem complex, in our opinion this complexity is well within the capabilities of analysts currently conducting standard CEA. The key to expanding the use of DCEA will be the development of better methods for assisting decision makers to clarify and quantify the nature of their inequality concerns, and better ways of communicating findings to nonspecialist audiences.

## Supplementary Material

Supplementary material
