# Supplementary material for: Distributional Cost-Effectiveness Analysis: A Tutorial
Source: Med Decis Making. 2015 Apr 23;36(1):8–19. doi: 10.1177/0272989X15583266 (PMC4853814; doi:10.1177/0272989X15583266)
Supplement: Supplementary material [file DS_10.11770272989X15583266.pdf]

APPENDIX

Estimating the baseline health distribution

Data on LE by IMD quintile and sex is published directly by the Office of National Statistics (16). However, for the purposes of our analysis we also require the underlying mortality rates used to estimate these figures in order to incorporate them in the decision analytical model where all-cause mortality is separated from colorectal cancer specific mortality. Unfortunately, these underlying mortality rates are not available by IMD quintile groups. So to ensure we remain consistent between our baseline QALE distribution and QALE distributions associated with the various implementations of the BCSP produced by our model, we use ONS mortality rates by social class (17) to proxy those by IMD, and apply the mapping between social classes and IMD quintile groups given in Table A.I.

Table AI Mapping between IMD Quintile Groups and Social Class

Deprivation (IMD Quintile)	Social Class
Q1 (Least Deprived)	I&II (Professional occupations & Managerial and technical occupations)
Q2	I&II (Professional occupations & Managerial and technical occupations)
Q3	IIIN (Skilled non-manual occupations)
Q4	IIIM (Skilled manual occupations)
Q5 (Most Deprived)	IV&V (Partly-skilled occupations & Unskilled Occupations)

We then use these mapped mortality rates to calculate the LE at birth by IMD quintile groups (2002-05) using the standard ONS methodology (18). Table A.II compares life expectancies estimated indirectly using the mapping process described above with published direct estimates of life expectancy by IMD quintile for the same period (2002-05). We see from the comparison that while the mapped values are on the whole reasonably close to the published values, they begin to diverge for the more deprived areas.

Table AII Comparison between Mapped and Published LE by IMD Quintile Group

Sex	Deprivation (IMD Quintile)	LE by Mapped IMD Quintiles (years)	LE Published IMD Quintiles (years)	Difference (Mapped – Published)
Male	Q1 (Least Deprived)	80.4	80.0	0.4
	Q2	80.4	78.6	1.8
	Q3	79.2	77.3	1.9
	Q4	77.7	75.4	2.3
	Q5 (Most Deprived)	76.2	72.2	4.0
Female	Q1 (Least Deprived)	83.7	83.2	0.5
	Q2	83.7	82.3	1.4
	Q3	82.6	81.5	1.1
	Q4	81.1	80.1	1.0
	Q5 (Most Deprived)	80.3	77.9	2.4

We next adjust these life expectancies for morbidity. To do this we adjust for age and sex by applying the relevant weights from the published EQ-5D Norms (19) for each age range (reproduced in Table A.III) and aggregate to give and age and sex adjusted QALE. Taking the example of a male in the least deprived IMD quintile group (Q1) we can read from Table A.II that their estimated life expectancy is 80.4 years. Using the weights in Table A.III we estimate the QALE for individuals in this subgroup as:

*24*0.94 + (35-25)*0.93 + (45-35)*0.91 + (55-45)*0.84 + (65-55)*0.78 + (75-65)* 0.78 + (80.5-75)*0.75 = 69.8 QALYs*

Table AIII QALY Weights by Age and Sex Based on EQ-5D Norms

Age	Male	Female
0-25	0.94	0.94
25-34	0.93	0.93
35-44	0.91	0.91
45-54	0.84	0.85
55-64	0.78	0.81
65-74	0.78	0.78
75+	0.75	0.71

In addition to quality adjusting LE for age and sex, we also would like to adjust for variation in quality of life by area level deprivation. In order to do this we turn to the ONS data for LE and disability free life expectancy (DFLE) by IMD quintile (16). We assume that the average quality adjustment we have applied by using the age and sex weights captures the adjustment for the middle IMD quintile group (Q3)for each sex, and calculate relative adjustment factors for the other IMD quintile groups by further assuming the ratio of DFLE to LE is the same as the ratio of QALE to LE. We use this data to calculate the adjustment factors shown in Table A.IV.

Table AIV Using LE and DFLE to Calculate QALE Adjustment Factors by IMD

Sex	Deprivation (IMD Quintile)	LE	DFLE	Ratio DFLE/LE	QALE Adjustment Factor
Male	Q1 (Least Deprived)	80.0	67.3	0.84	1.03
	Q2	78.6	64.3	0.82	1.00
	Q3	77.3	63.4	0.82	1.00
	Q4	75.4	59.7	0.79	0.96
	Q5 (Most Deprived)	72.2	54.2	0.75	0.91
Female	Q1 (Least Deprived)	83.2	67.8	0.81	1.02
	Q2	82.3	65.7	0.80	1.00
	Q3	81.5	64.9	0.80	1.00
	Q4	80.1	61.8	0.77	0.97
	Q5 (Most Deprived)	77.9	57.2	0.73	0.92

Applying the adjustment factor to our QALE estimate for our male from IMD Q1 gives a refined QALE estimate taking into account area level deprivation of:

*69.8 * 1.03 = 72 QALYs*

Similar calculations for the other subgroups yield the QALE estimates in Table A.V.

Table AV QALE by Sex and Deprivation

Sex	Deprivation (IMD Quintile)	QALE
Male	Q1 (Least Deprived)	72.2
	Q2	70.5
	Q3	69.1
	Q4	66.6
	Q5 (Most Deprived)	60.2
Female	Q1 (Least Deprived)	74.8
	Q2	73.1
	Q3	71.8
	Q4	69.2
	Q5 (Most Deprived)	63.2

Ordering the subgroups by QALE from least healthy to most healthy and adjusting for the size of each subgroup we are able to create a population distribution of QALE at birth taking into account differential mortality and morbidity by age, sex and area level deprivation.

REFERENCES

16. ONS. Inequalities in disability-free life expectancy by area deprivation: England, 2001-04 to 2007-10 [Internet]. 2013. Available from: http://www.ons.gov.uk/ons/rel/disability-and-health-measurement/sub-national-health-expectancies/inequality-in-disability-free-life-expectancy-by-area-deprivation--england--2003-06-and-2007-10/rft-inequalities-in-disability.xls

17. ONS. Longitudinal Study age-specific mortality data 1972-2005 (supplementary tables) [Internet]. 2007. Available from: http://www.ons.gov.uk/ons/rel/health-ineq/health-inequalities/trends-in-life-expectancy-by-social-class-1972-2005/longitudinal-study-age-specific-mortality-data-1972-2005--supplementary-tables-.xls

18. Johnson B, Blackwell L. Review of methods for estimating life expectancy by social class using the ONS Longitudinal Study. 2007 p. 28–36.

19. Kind P, Hardman G, Macran S. UK population norms for EQ-5D [Internet]. 1999 [cited 2012 Oct 31]. Available from: http://www.york.ac.uk/media/che/documents/papers/discussionpapers/CHE Discussion Paper 172.pdf
